# Efficacy of Transcutaneous Pulsed Radiofrequency Treatment in Subacromial Impingement Syndrome: A Randomized Controlled Study

**DOI:** 10.3390/jcm13237462

**Published:** 2024-12-07

**Authors:** Ayça Utkan Karasu, Ayza Kılıç, Belgin Karaoğlan

**Affiliations:** Department of Physical Medicine and Rehabilitation, Faculty of Medicine, Gazi University, 06500 Ankara, Turkey; dr.ayzamamedova@gmail.com (A.K.); belginkaraoglan@gmail.com (B.K.)

**Keywords:** shoulder pain, subacromial impingement syndrome, transcutaneous pulsed radiofrequency

## Abstract

**Background:** This study assessed Transcutaneous Pulse Radiofrequency Therapy’s (TCPRF) effectiveness in reducing shoulder pain and improving shoulder function. **Methods:** A double-blind randomized controlled trial involved 50 SAIS patients with chronic shoulder pain. Participants were randomized into two groups: the study group (n = 27) received TCPRF, while the control (n = 23) received sham treatment. The primary outcome was shoulder pain, secondary outcomes, including shoulder function, quality of life, and range of motion, were assessed at baseline, and at 1, 4, and 12 weeks using the Visual Analog Scale (VAS), Shoulder Pain and Disability Index (SPADI), and the SF-36 questionnaire. Supraspinatus tendon thickness (SSPT) and acromiohumeral distance (AHD) were measured by ultrasound. **Results:** Both groups showed reductions in activity and resting pain over 12 weeks. In the control, activity pain dropped from a median (IQR) of 8 (7–8) to 6 (3–7), and in TCPRF from 8 (7–10) to 3.5 (2–6.3), with no significant difference at 12 weeks (*p* = 0.192). Resting pain decreased from 3 (1–6) to 1 (1–3) in the control, and from 3 (2–4) to 0 (0–2) in TCPRF, showing a lower resting pain at 12 weeks (*p* = 0.041). SPADI-Total scores decreased from 87 (54–105) to 50 (29–82) in the control and from 84 (69–107) to 21 (9–66.3) in TCPRF, favoring TCPRF at 12 weeks (*p* = 0.017). SPADI–Disability scores reduced from 49 (30–63) to 30 (15–30) in control and from 47 (35–62) to 11 (5.8–38.8) in TCPRF, also favoring TCPRF (*p* = 0.008). Both groups showed similar improvements in other outcomes. **Conclusions:** TCPRF reduced resting pain and shoulder disability in SAIS over 12 weeks, though other outcomes showed similar improvement in both groups. Further studies are needed to determine long-term effects.

## 1. Introduction

Shoulder pain ranks among the most prevalent musculoskeletal issues, closely following low back and neck pain [1]. Its lifetime occurrence varies widely, ranging from 6.7% to 66.7% [2]. This condition poses a significant medical and socioeconomic challenge, impacting both individuals and society. It can lead to workforce reduction and hinder daily activities. Notably, shoulder pain often persists, with approximately 40% of individuals reporting ongoing complaints even after seeking medical attention for up to 12 months [3]. Subacromial impingement syndrome (SAIS) contributes to about 44–65% of physician referrals for shoulder pain [4]. SAIS involves symptoms and clinical signs stemming from pathologies affecting the rotator cuff tendon or adjacent structures, causing a narrowing of the space between the acromion and the humeral head [4]. This syndrome encompasses various conditions within the subacromial space, including partial-thickness rotator cuff tears, rotator cuff tendinosis, calcific tendinitis, and subacromial bursitis [5]. SAIS presentation can vary significantly due to individual anatomical and biomechanical factors. Variations in acromial shape, rotator cuff morphology, and glenohumeral alignment, as well as differences in shoulder movement patterns, can influence the degree of subacromial impingement and response to treatment. These factors may contribute to the variability observed in treatment outcomes among patients with SAIS [5].

Subacromial impingement syndrome differs from other shoulder pathologies in its pathophysiology and its specific response to targeted subacromial treatments. Given its localized nature within the subacromial space, interventions that expand the acromiohumeral distance or address inflammation in this area are particularly effective for SAIS, distinguishing it from conditions such as adhesive capsulitis or rotator cuff tears, which may require alternative therapeutic approaches. Various methodologies can be employed in the management of SAIS. Treatment modalities encompass not only physical interventions such as stretching, range of motion exercises, and strengthening regimens targeting the periscapular muscles and the rotator cuff, as recommended by extant literature [6], but also extend to conservative approaches. These non-exercise-based interventions encompass transcutaneous electrical nerve stimulation (TENS) [7], microwave diathermy therapy [8], ultrasound therapy, low-level laser therapy [9], radial extracorporeal shockwave therapy [10], acupuncture therapy [11], pulsed electromagnetic field therapy [12], manual therapy [13], kinesio taping therapy [14], and localized drug injection involving corticosteroids, hyaluronate, or nonsteroidal anti-inflammatory drugs (NSAIDs) [9,15,16]. Surgery for SAIS, including procedures like subacromial decompression, has been a common treatment approach, especially when conservative treatments have failed to relieve symptoms. However, the evidence supporting its effectiveness is mixed and has been the subject of considerable debate in recent years. Several high-quality studies have questioned the efficacy of surgical interventions for SAIS. It was demonstrated that the outcomes of surgical treatments may not significantly differ from those of non-surgical treatments or placebo in terms of pain relief, improvement in function, and quality of life. Current guidelines suggest that surgical treatment of SAIS should be considered for patients who do not respond to non-operative treatments and are not suitable candidates for further rehabilitation therapies [17]. Therefore, the search for new treatments that could be effective in the treatment of SAIS is valuable.

The selection of non-exercise-based conservative treatments for SAIS management was guided by their potential to reduce key symptoms associated with this condition, such as pain, inflammation, edema, and limitations of the range of motion. Although conservative treatments often share common goals, each method offers unique mechanisms of action that address specific aspects of SAIS. TCPRF provides anti-inflammatory and analgesic effects suitable for SAIS. A noninvasive treatment approach introduced in 2004 is Transcutaneous Pulse Radiofrequency Therapy (TCPRF). TCPRF is a needle-free, painless, outpatient method that does not require anesthesia, sedation, or the recovery period typically associated with surgical treatments. Unlike surgical procedures, which often necessitate a recovery period to allow for tissue healing and functional restoration, TCPRF can be performed without the need for any post-treatment recovery, making it a convenient alternative for patients seeking noninvasive relief [18]. It is believed that TCPRF treatment works by utilizing the electric and magnetic fields generated by radiofrequency signals transmitted through superficially placed gel electrodes on the affected area [19]. The precise mechanism of action underlying TCPRF therapy, employed to enhance treatment efficacy, remains poorly understood [20]. Proposed mechanisms of action for pulsed radiofrequency therapy (PRFT), a method in use longer than TCPRF, include the induction of heat lesions, electroporation, electric field effects, magnetic field effects, and modulation of the inflammatory cytokines involved in immune response [1]. An alternating electric field of appropriate strength and speed can alter cell membrane functions and disrupt the synaptic transmission of A-Delta and C fibers [1]. Weaker electric field effects may persistently suppress synaptic transmission, antagonizing the long-term potentiation seen in chronic pain conditions [20]. Additionally, PRFT has demonstrated its ability to reduce inflammatory cytokines like IL-1 beta, TNF-alpha, and IL-6, consequently decreasing C-reactive protein levels [19]. Recent studies have provided insights into the mechanisms by which pulsed radiofrequency (PRF) exerts its therapeutic effects. Experimental findings indicate that PRF can enhance TNF-α secretion in monocytes [21], stimulate the expression of immune-modulating and anti-inflammatory genes in astrocytes [22], and potentially reverse oxidative stress, likely through the influence of PRF’s magnetic field component [23]. This anti-inflammatory action aligns with current theories that suggest PRF’s efficacy in treating inflammatory conditions by modulating immune responses and restoring redox balance. These effects of PRF support its potential role in reducing pain and inflammation in musculoskeletal conditions such as SAIS.

In recent years, ultrasonography has become a frequently used noninvasive method of diagnosing and monitoring musculoskeletal pathologies [24]. A reduction in the acromiohumeral distance (AHD), coupled with thickening of the supraspinatus tendon (SSPT), can contribute to SAIS [25]. The AHD is typically measured as the shortest distance between the inferior aspect of the acromion and the humeral head, with reductions in this space indicative of potential impingement. Similarly, SSPT thickness is assessed by measuring the distance across the tendon near its insertion point, where increased thickness can signal tendon pathology associated with SAIS [25]. Ultrasonographic measurements of AHD and SSPT thickness have been shown to be reliable and effective diagnostic and follow-up tools for subacromial impingement syndrome [26].

Limited literature exists on the application of TCPRF to various painful musculoskeletal conditions, including in the shoulder and knee joints [18,19,27]. This study aims to evaluate the effect of TCPRF treatment on pain, disability, range of motion, quality of life, and, for the first time, ultrasonographic measurements in patients diagnosed with SAIS.

## 2. Materials and Methods

### 2.1. Study Design

This double-blind, randomized controlled trial was conducted at a university hospital from March 2020 to August 2021. Fifty participants with shoulder pain and SAIS were recruited after providing written informed consent. The study adhered to the Declaration of Helsinki, was approved by the Gazi University ethics committee (Decision number: 2020-26) and registered on clinicaltrials.gov (ID: NCT04289610).

Participants’ eligibility was determined through detailed anamnesis, physical examination, and ultrasonographic assessment. Participants were assessed before treatment and at the 1st, 4th, and 12th weeks post-treatment, with evaluations including the Visual Analog Scale (VAS) for pain, the Shoulder Pain and Disability Index (SPADI), Short Form-36 (SF-36), shoulder range of motion (ROM), and ultrasonographic SSPT and AHD measurements. All of the assessments were conducted by the same evaluator, who was blinded to the group assignments.

### 2.2. Participants

Eligible participants, aged 18–65, had shoulder pain lasting over three months and positive results in at least three of five clinical tests (Neer, Hawkins–Kennedy, painful arc, Jobe, and external rotation resistance) [28]. They were required to have not received shoulder pain treatment in the past three months. Exclusion criteria included systemic rheumatic diseases, malignancy, infections, adhesive capsulitis, full-thickness supraspinatus tears, cervical radiculopathy, prior shoulder and neck surgeries, pregnancy, and pacemaker use.

### 2.3. Blinding Process

The evaluator and participants remained blinded to the administered treatment until the 12th week evaluation of the last patient. Unblinding occurred after statistical analysis. In our study, TCPRF treatment settings were imperceptible, and participants were informed that they were not expected to feel any sensations during the procedure. The treatment was administered individually to each participant to prevent interactions or information exchange. Number sets were generated using a computer program (https://stattrek.com/statistics/random-number-generator, accessed on 15 March 2020), which created pseudo-random numbers based on an initial seed, ensuring independent allocation without a discernible pattern. Allocation concealment was achieved through consecutively numbered, 56 sealed envelopes, opened only by the physiotherapist immediately before the treatment assignment. This physiotherapist did not participate in the evaluation, ensuring adherence to the double-blind study protocol. Simple randomization was applied without stratification, as was deemed appropriate for the sample size and study design.

### 2.4. Interventions

A trained physiotherapist administered TCPRF therapy using a radiofrequency device, following Taverner et al.’s protocol [19].

**Figure 1:**
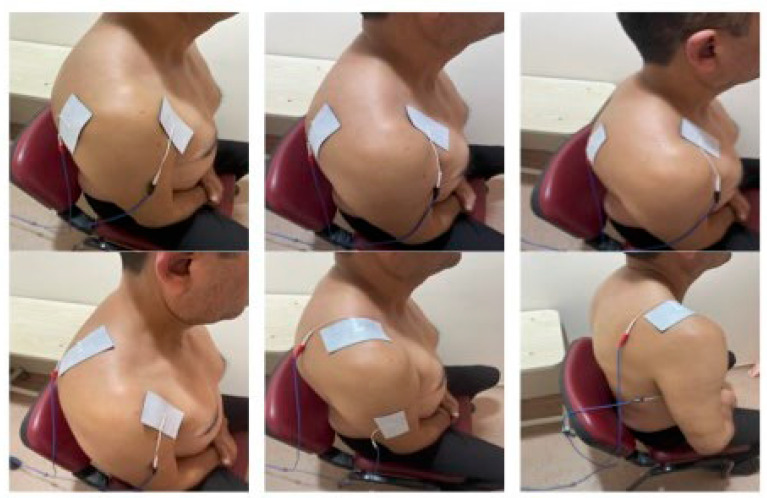
Shows six electrode pair positions.

Electrodes were placed in six standardized areas on the painful shoulder for both groups, with the active group receiving 80 V and the sham group receiving 0 V (Figure 1). In the anteroposterior plane, they were placed laterally and medially. In the oblique plane, one electrode was positioned from the deltopectoral triangle to the posterior shoulder joint, targeting below the spine of the scapula, while another was placed from the anterior shoulder joint to the supraspinatus, targeting above the spine of the scapula. In the coronal plane, electrodes were placed from the acromioclavicular joint to the deltoid insertion on the humerus and from the acromioclavicular joint to the inner humerus, extending just below the hairline in the axilla. TCPRF was applied at each site for 2 min, with a frequency of 5 Hz and a pulse duration of 10 ms, each TCPRF treatment session lasted approximately 15 min. The treatment utilized a Top TLG-10 STP radiofrequency lesion generator (Equip-Medikey, Gouda, The Netherlands) and 45 × 98 mm disposable electrodes (FIAB S.p.A, Florence, Italy), administered in a single session for both groups.

During the three-month follow-up, participants were educated on modifying and avoiding overhead activities. Therapeutic exercises, prescribed by a blinded researcher, were recommended to all, and analgesic usage was documented as needed in both groups.

### 2.5. Outcome Measures

Visual Analog Scale: The primary outcome, shoulder pain, was measured using the VAS during activity and rest. Participants were instructed to indicate their shoulder pain severity at the time of evaluation by marking a position along a 100 mm long line. In the VAS, 0 represented no pain, while 10 indicated the most severe pain imaginable [29].

Shoulder Pain and Disability Index: Shoulder disability was evaluated using the Shoulder Pain and Disability Index (SPADI), a self-administered 13 item questionnaire that consists of two dimensions: a 5-item subscale measures pain, and an 8-item subscale measures disability. Participants were asked to score how much pain and difficulty they had during physical activities in the past week, with zero indicating no pain or difficulty and ten indicating inability to perform the activity without help. Scores can range from 0 to 50 on the pain scale and from 0 to 80 on the disability scale. Total scores range from 0 to 130, with zero indicating no difficulty and 130 indicating maximum disability [30].

Short Form 36 Health Survey Questionnaire: Quality of life was assessed using the Short Form 36 Health Survey Questionnaire (SF-36), a 36-item self-report measure of health-related quality of life. It is a reliable and valid questionnaire for musculoskeletal diseases, featuring eight subscales measuring different domains of health-related quality of life: physical functioning, role-physical, bodily pain, general health, vitality, social functioning, role-emotional, and mental health. Each scale is directly transformed into a 0–100 scale. Lower scores indicate greater disability, while higher scores across all subscales represent better health and functioning [31].

Shoulder range of motion was measured using a goniometer, assessing flexion, extension, abduction, adduction, internal rotation, and external rotation of the shoulder joint [32].

Ultrasonographical measurements: To monitor the effectiveness of the treatment, AHD and supraspinatus tendon thickness were measured ultrasonographically by a single investigator with 3 years of experience in musculoskeletal ultrasonography. Prior to initiating measurements, the investigator conducted pilot ultrasound exams to ensure competence and repeatability. Ultrasound examinations were performed using a GE Logiq P5 US scanner equipped with a 6–18 MHz linear-array transducer (GE Healthcare, Wauwatosa, WI). For AHD measurement, participants were seated on a chair with their arms at their sides, elbows flexed to 90 degrees, and hands resting on their laps. The ultrasound transducer was placed longitudinally along the center of the acromion. Once the acromion and humerus were imaged, the transducer was advanced until the most anterior part of the acromion was visible along with a simultaneous clear image of the humeral head. AHD was measured as the shortest distance between the inferolateral corner of the anterior acromion and the humeral head, following a line parallel to the acoustic shadow formed by the acromion. The validity of this measurement protocol has been previously demonstrated [33]. To measure supraspinatus tendon thickness (SSPT), participants’ upper extremities were positioned in the modified Crass position, with the palms on the iliac crests and elbows pointing posteriorly. During the examination, the ultrasound transducer was placed on the acromion in the transverse plane and moved laterally to visualize the tendon. The transducer was then moved anteriorly until the intra-articular part of the long head of the biceps was visible, and the image was saved. Measurements were taken at 5 mm and 10 mm posterior to the biceps tendon corner, with the average used [34]. Analgesic use was recorded at the 1st, 4th, and 12th weeks.

### 2.6. Statistical Analysis

According to Cohen’s recommendations, typical statistical analyses aiming for an 80% power level (corresponding to a 20% chance of Type II error) at a 0.05 significance level (equivalent to a 95% confidence interval) are widely regarded as acceptable, tolerable, and infrequent occurrences of error [35]. Using G*Power software (Version 3.1.9.6) to conduct the power analysis, and considering the treatment outcomes observed in a prior clinical trial [19], we anticipated an effect size, as denoted by a customary Cohen’s d of 0.80, indicating 21 patients in each group, as the appropriate sample sizes for this study. To account for potential follow-up losses, we aimed to recruit a total of 50 subjects.

IBM SPSS 20.0 was used for the statistical analyses. Normality was tested with the Kolmogorov–Smirnov test. Numerical data were presented as median (IQR) and categorical data as frequencies. One-way ANOVA or Kruskal–Wallis tests compared numerical variables, and chi square or Fisher’s exact tests compared categorical data. VAS score changes were analyzed using the Friedman test, with post hoc pairwise comparisons via Wilcoxon signed-rank tests, applying Bonferroni corrections for multiple comparisons. Statistical significance was set at *p* < 0.05. The adjusted significance level was *p* < 0.0083. Intraobserver reliability for ultrasound measurements was assessed using intraclass correlation coefficient (ICC) analysis.

## 3. Results

**Figure 2:**
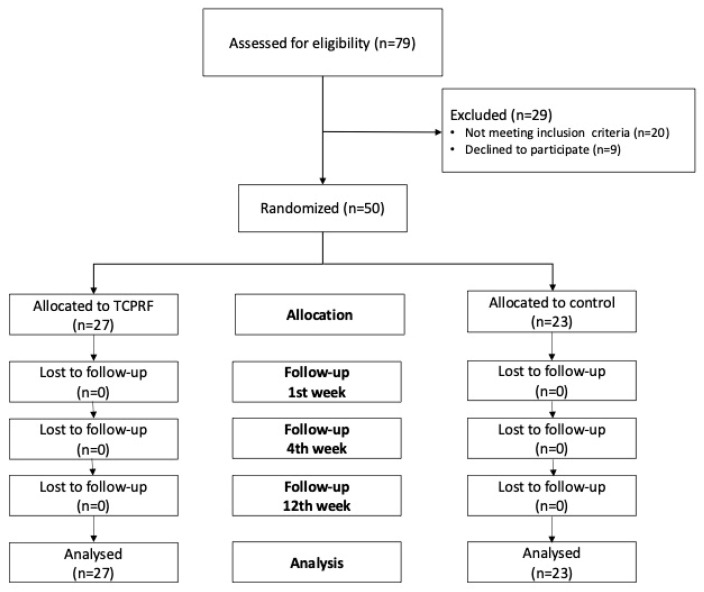
Study flowchart.

The study initially assessed 79 individuals for eligibility. However, nine individuals (31%) declined to participate, and an additional twenty did not meet the inclusion criteria and were consequently excluded. Among the excluded participants, six (21%) had received treatment for shoulder pain in the last three months, two (7%) had systemic rheumatic diseases, four (14%) had adhesive capsulitis, three (10%) had bicipital tendinitis, three (10%) had cervical radiculopathy, and two (7%) had a full-thickness tear of the supraspinatus tendon. The final sample consisted of 27 participants in the TCPRF group and 23 in the control group (Figure 2).

Baseline characteristics, including age, BMI, education level, affected shoulder, and duration of pain, were comparable between the two groups (Table 1). The mean age was 57.3 ± 6.8 years in the TCPRF group and 56.7 ± 7.1 years in the control group (*p* = 0.745). Full demographic and clinical variables are shown in Table 1.

Pain during activity (VAS) and SPADI–Pain scores were comparable between the groups at baseline, as well as at the 1st, 4th, and 12th weeks. Similarly, resting pain (VAS) showed no significant difference between the groups at baseline, the 1st week, and the 4th week. However, by the 12th week, a significant difference emerged between the control and TCPRF groups (*p* = 0.041). SPADI–Total scores were also comparable between the groups at baseline, the 1st week, and the 4th week, but a significant difference was observed at 12 weeks (*p* = 0.017). Likewise, SPADI–Disability scores were similar across the groups at baseline, the 1st week, and the 4th week, with a significant difference appearing at the 12th week (*p* = 0.008) (Table 2).

**Figure 3:**
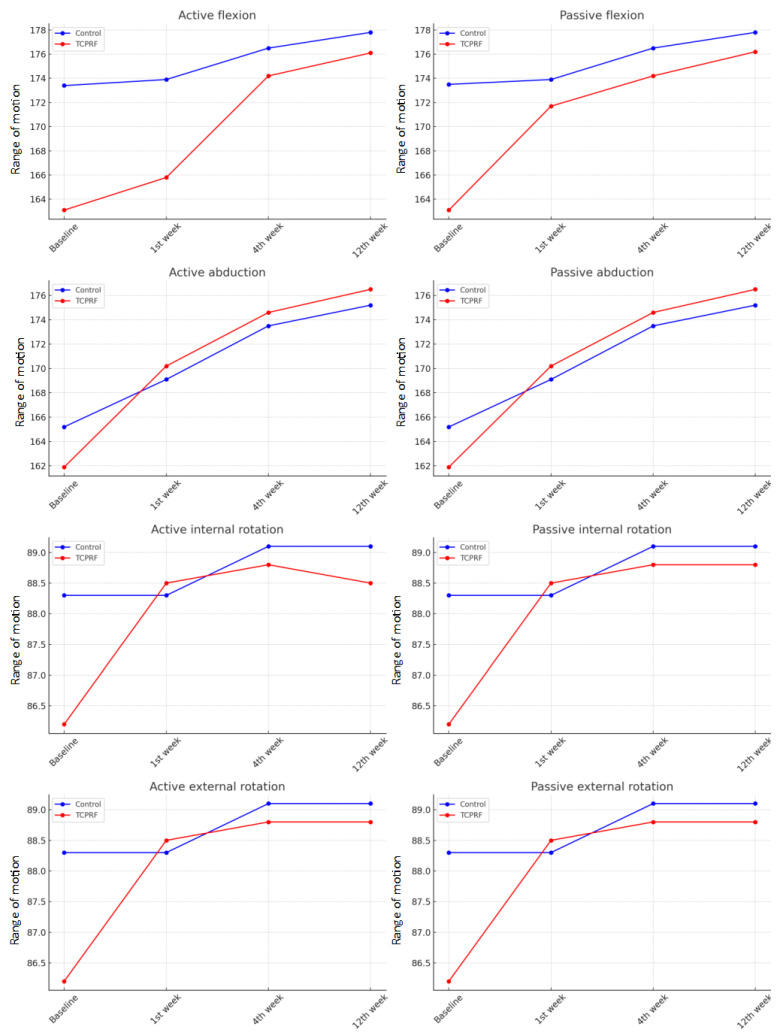
Shoulder range of motion measurements.

The measurements of active and passive flexion, abduction, internal rotation, and external rotation angles on the affected shoulders of the participants were assessed. In the initial evaluation (baseline), both active and passive flexion, abduction, internal rotation, and external rotation angles were similar between the two treatment groups (*p* > 0.05). Throughout the study period, active and passive flexion, abduction, internal rotation, and external rotation angles remained similar between the groups at the first, 4th, and 12th weeks (*p* > 0.05) (Figure 3).

**Figure 4:**
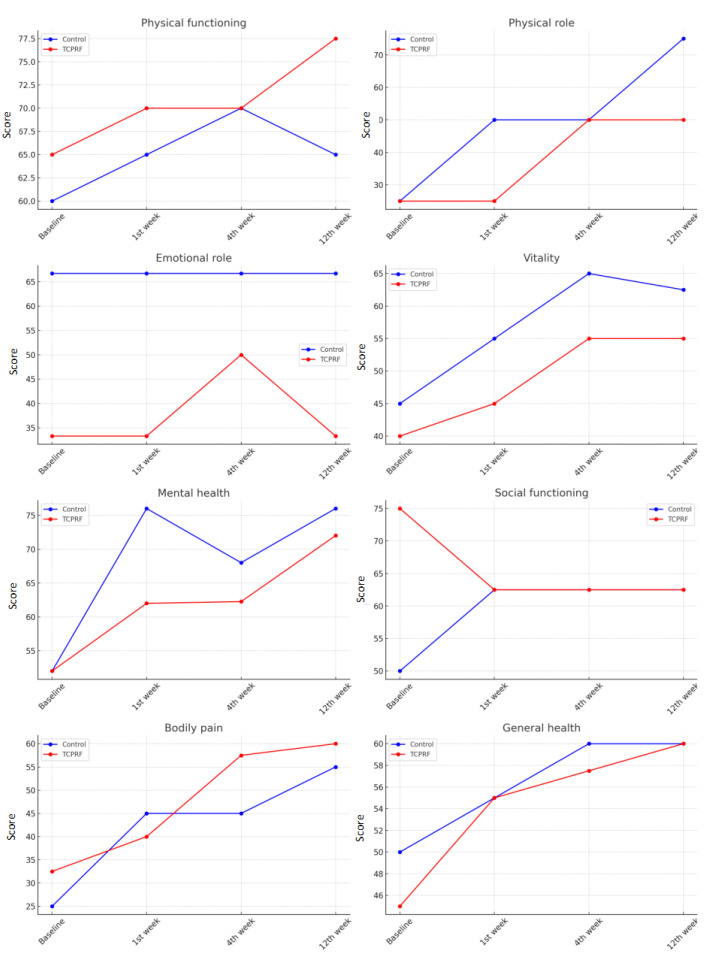
Short Form 36 Health Survey Questionnaire results.

The evaluation of health-related quality of life using the SF-36 questionnaire in both the control and study groups showed similar scores for physical functioning, physical role, emotional role, vitality, mental health, social functioning, pain, and general health perception at baseline, week 1, week 4, and week 12 (*p* > 0.05). This suggests that the two groups had similar quality of life measures across these domains before and during the study (Figure 4).

The ultrasound measurements were conducted twice for each patient, with a one-week interval, by the same evaluator on the unaffected shoulder to assess intra-rater repeatability. The intra-rater reliability analysis for consecutive measurements of AHD and SSPT demonstrated a high intraclass correlation coefficient (ICC = 0.801, 95% CI: 0.675–0.883; *p* < 0.001 for AHD; ICC = 0.839, 95% CI: 0.733–0.906; *p* < 0.001 for SSPT), indicating good reliability.

At the 12th week, SSPT was 6.6 mm (4.8–7.7 mm) in the TCPRF group and 6.5 mm (5.6–7.9 mm) in the control group (*p* = 0.952). Both groups experienced significant reductions over time, with baseline medians of 7.1 mm (4.8–9.0 mm) in TCPRF and 6.9 mm (5.6–7.9 mm) in the control group (TCPRF: χ^2^(3) = 8.059, *p* < 0.045 and control: χ^2^(3) = 10.479, *p* < 0.015 respectively).

For AHD, baseline median values were 11 mm (10–11) in TCPRF and 10.2 mm (8.8–10.6) in the control group (*p* = 0.063), showing no significant difference between the groups.

Analgesic use was assessed at three time points: baseline to the 1st week, 1st to 4th weeks, and 4th to 12th weeks. The percentage of patients not using analgesics increased over time in both groups. From baseline to the 1st week, 69.6% of the control group and 70.4% of the TCPRF group reported no analgesic use (*p* = 0.373). By the 4th week, this percentage rose to 82.7% in the control group and 81.5% in the TCPRF group (*p* = 0.889). By the 12th week, 86.9% of the control group and 88.9% of the TCPRF group reported no analgesic use (*p* = 0.978) (Table 3).

Significant changes were observed over time within both the control and TCPRF groups for several outcomes. Regarding activity pain, there was a significant reduction in both the control group (χ^2^(3) = 30.138, *p* < 0.001) and the TCPRF group (χ^2^(3) = 37.843, *p* < 0.001). Similarly, resting pain decreased significantly in the control group (χ^2^(3) = 8.953, *p* = 0.03) and the TCPRF group (χ^2^(3) = 19.420, *p* < 0.001). The SPADI–Total, SPADI–Pain, and SPADI–Disability scores also showed significant improvements in both groups: control (χ^2^(3) = 28.208, *p* < 0.001; χ^2^(3) = 25.227, *p* < 0.001; χ^2^(3) = 20.620, *p* < 0.001, respectively) and TCPRF (χ^2^(3) = 40.344, *p* < 0.001; χ^2^(3) = 43.135, *p* < 0.001; χ^2^(3) = 35.020, *p* < 0.001, respectively).

Significant improvements were observed over time in both active and passive flexion across the groups. In the control group, improvements in active flexion (χ^2^(3) = 8.489, *p* = 0.037) and passive flexion (χ^2^(3) = 8.489, *p* = 0.037) were noted. Similarly, in the TCPRF group, significant improvements were found in active flexion (χ^2^(3) = 18.341, *p* < 0.001) and passive flexion (χ^2^(3) = 20.049, *p* < 0.001).

For abduction, both active and passive measures showed significant improvements. In the control group, active abduction (χ^2^(3) = 18.000, *p* < 0.001) and passive abduction (χ^2^(3) = 18.000, *p* < 0.001) improved significantly. The TCPRF group also exhibited significant gains in active abduction (χ^2^(3) = 20.179, *p* < 0.001) and passive abduction (χ^2^(3) = 20.179, *p* < 0.001).

Regarding internal and external rotation, significant improvements were only observed in the TCPRF group. Active and passive internal rotation both improved (χ^2^(3) = 9.273, *p* = 0.026), as did active and passive external rotation (χ^2^(3) = 9.273, *p* = 0.026).

In terms of quality of life, significant changes were observed over time within both the control and TCPRF groups for several outcomes. In the control group, physical functioning improved (χ^2^(3) = 8.595, *p* = 0.035), and the TCPRF group showed similar improvements (χ^2^(3) = 10.939, *p* = 0.012). For physical role, significant improvements were only observed in the control group (χ^2^(3) = 22.754, *p* < 0.001). Vitality scores also improved significantly in both groups, with the control group (χ^2^(3) = 19.095, *p* < 0.001) and the TCPRF group (χ^2^(3) = 22.291, *p* < 0.001) showing notable gains. Mental health improvements were seen in both the control (χ^2^(3) = 9.210, *p* = 0.027) and TCPRF (χ^2^(3) = 11.086, *p* = 0.011) groups. For bodily pain, both groups demonstrated significant reductions, with the control group (χ^2^(3) = 15.623, *p* = 0.001) and the TCPRF group (χ^2^(3) = 23.090, *p* < 0.001) showing improvements. In terms of general health, significant improvements were only observed in the TCPRF group (χ^2^(3) = 11.371, *p* = 0.010).

Tables of multiple comparisons for changes in activity and rest pain, SPADI scores, ROM, SF-36, and SSPT measurements over time are summarized in Table 4.

## 4. Discussion

In our study, the application of TCPRF treatment for SAIS demonstrated limited evidence to suggest superiority over the sham group. Both groups experienced a reduction in activity pain, rest pain, and SPADI scores over the 3-month study period. Although the resting pain score and SPADI–Total and SPADI–Disability scores were lower in the TCPRF group at the end of the 12th week, the TCPRF treatment’s superiority regarding activity-related pain could not be established. Furthermore, at time points other than the 12th week, there was no discernible difference between the two groups in any of the outcome measures. Both groups showed significant improvements in shoulder range of motion and quality of life evaluations. However, there were no significant differences observed between the two groups at the 1st, 4th, and 12th week assessments.

SAIS is a prevalent cause of shoulder pain, often managed with a combination of medical treatment and exercises. The precise mechanism of action underlying TCPRF therapy, employed to enhance treatment efficacy, remains poorly understood. Given the settings used in our study, TCPRF’s effectiveness may be attributed to mechanisms associated with electric field changes, independent of temperature. Currently, there is a limited body of literature investigating the impact of TCPRF treatment on shoulder pain. For instance, Korkmaz et al. compared PRFT applied to the suprascapular nerve with conventional transcutaneous electrical nerve stimulation in patients with chronic shoulder pain [36]. This randomized controlled trial, which involved forty patients suffering from chronic shoulder pain, evaluated VAS, range of motion, SPADI, and SF-36 scores before treatment and at weeks 1, 4, and 12. The study found no significant differences between groups, except for the SPADI–Total score. Similarly, our study showed no significant differences between the treatment and control groups, except for the resting pain, SPADI–Total, and SPADI–Disability scores at the 12th week. However, direct comparison is challenging, as Korkmaz et al.’s PRF treatment was delivered via needle rather than transcutaneously. In a pilot study, TCPRF was compared with TENS for the treatment of chronic shoulder tendinitis, involving fifty patients over three months [20]. The study assessed comfort levels, adverse events, Constant–Murley Shoulder (CMS) scores, and pain, enjoyment of life, and general activity (PEG) scores, finding no adverse events and similar tolerability between treatments. Notably, the TCPRF group exhibited superiority over the TENS group in terms of PEG score and CMS. Several factors may account for the improved results of TCPRF treatment compared to our study. First, the outcome measures differed between the studies. Second, TCPRF was administered three times in the pilot study, whereas our study used it only once, which could affect efficacy. Third, the pilot study included patients with shoulder tendinosis, while our study focused solely on SAIS, potentially influencing the different outcomes. Taverner et al. conducted a randomized controlled trial of 51 patients scheduled for shoulder surgery [19]. They compared TCPRF treatment with a sham treatment, evaluating VAS pain scores, Aggregate Brief Pain Inventory pain (BPI-p) scores, BPI pain interference with function (BPI-pif) scores, Oxford Shoulder Score (OSS), and shoulder range of motion before treatment, at 4 weeks, and at 12 weeks. The TCPRF group exhibited significant improvements in the night, activity VAS pain scores, and BPI-p scores, which assess pain, as well as the BPI-pif scores and OSS, indicating functional improvements. In contrast, while our study found statistically significant improvements in both groups, there was no significant difference between the TCPRF and control groups except for resting pain, SPADI–Total, and SPADI–Disability scores at 12 weeks. It is important to note that Taverner et al.’s study included patients with severe conditions like frozen shoulder and full-thickness rotator cuff tears, whereas our study involved less severe cases with no instances of frozen shoulder or full-thickness tears. Additionally, the patient education and therapeutic exercises provided in our study could have led to improvements in both groups.

The differential improvement observed in the physical role subscale of the SF-36, where significant gains were noted only in the control group, merits consideration. The physical role subscale assesses the extent to which physical health limitations impact an individual’s daily responsibilities and occupational tasks, such as reduced activity performance, limited working hours, and diminished efficiency. Unlike parameters that directly measure pain relief or functional range, physical role reflects a more comprehensive sense of physical capacity in daily life. Given its broader scope, changes in physical role limitations may not be immediately apparent, as individuals generally require sustained improvement in pain and specific joint function to perceive gains in overall functional roles. Therefore, a more extended follow-up period might be necessary to fully reveal its impact on physical role limitations, reflecting potential long-term enhancements in everyday functional capacity.

Recent studies indicate that the normal AHD in a neutral shoulder position ranges from 9 mm to 12 mm. In individuals with tendon pathology, AHD measurements tend to decrease, typically ranging from 6 mm to 10 mm [11,25,37]. In our study, the median AHD measured 10.3 mm, which is near the upper limit of the AHD values reported for patients with SAIS. Given that SAIS is a dynamic pathology that may worsen with certain shoulder positions, dynamic ultrasound measurements of AHD could provide a more accurate assessment of impingement. Also, research has shown that SSPT is typically greater in the symptomatic shoulder compared to the asymptomatic shoulder in cases of SAIS [34,38]. Our study also noted an increase in tendon thickness on the affected side, although this difference was not statistically significant. Additionally, ultrasonographic assessments revealed a decrease in tendon thickness over time in both the treatment and control groups, which aligned with clinical improvements. However, acromiohumeral distance (AHD) and supraspinatus tendon thickness (SSPT) did not show significant differences between the groups. Several factors may have influenced these findings. AHD was assessed only at baseline, and no follow-up measurements were taken to track changes over time. Regarding SSPT, while both groups exhibited improvements in tendon thickness over time, no significant differences were observed between the groups. This might be due to the single-session design of the intervention, which may not have been sufficient to induce structural changes detectable through ultrasonography. Future studies with multiple-session TCPRF protocols would allow for a better understanding of how TCPRF affects these measures and whether cumulative sessions might lead to more pronounced changes.

The strengths of our study include its randomized controlled design, the presence of a control group, and repeated measurements. To date, only one randomized controlled trial investigating the efficacy of transcutaneous pulsed radiofrequency treatment for patients with shoulder pain scheduled for surgery has been published in the literature [19]. Furthermore, our study exclusively focused on patients with a specific SAIS, unlike previous studies that included a heterogeneous group of shoulder pain conditions, thus minimizing the influence of varied diagnoses on treatment outcomes. We evaluated patients across multiple dimensions—pain, disability, quality of life, and joint range of motion. Notably, our study is the first to use ultrasound to assess pathologies during TCPRF treatment, showing improvements in ultrasonographic findings alongside symptom reduction.

Our study faced several limitations. First, all of the participants received uniform education on SAIS, therapeutic exercises, and, if needed, analgesics, which might have improved outcomes across both groups, possibly masking differences between them. Ethical considerations necessitated providing this standard of care to all participants. To address this limitation, both cohorts received identical standard training and exercise regimens. Additionally, we meticulously documented patient analgesic requirements and the types of analgesics administered. Given the comparability in analgesic utilization between the two groups, and notably the limited reliance on analgesics by a small number of patients, it is reasonable to infer that analgesic usage may have had a negligible impact on outcomes. The observed analgesic effect in the sham group at the 1st and 4th-week assessments warrants consideration of several factors. One potential explanation is the influence of the therapeutic exercises provided to both groups, as these exercises are known to alleviate pain and improve function by enhancing musculoskeletal support and reducing inflammation. Another explanation could be that the therapeutic effects of TCPRF emerge more clearly over time, which aligns with the more pronounced improvements in pain and disability observed in the TCPRF group by the 12th week. This delayed response suggests that while placebo and exercise effects are immediate, TCPRF may provide additional, sustained therapeutic benefits. Consequently, isolating TCPRF’s effect becomes challenging, and future studies may benefit from a control group that does not receive this standard care.

Additionally, SAIS is a heterogeneous condition with various underlying pathologies, which might influence TCPRF’s efficacy differently across subacromial space pathologies. Our study employed a single-session TCPRF application, and significant improvements have been observed with this approach in previous research. However, multiple TCPRF sessions could yield more pronounced therapeutic effects. Thus, the single-session design may have limited TCPRF’s effectiveness. Future research should explore whether a multi-session TCPRF protocol enhances outcomes for SAIS patients and investigate how different underlying pathologies contributing to SAIS might respond to TCPRF treatment.

In our study, TCPRF demonstrated selective efficacy in certain parameters, such as resting pain and SPADI scores. This may suggest that TCPRF is more effective in alleviating pain types that are more closely related to inflammatory processes, such as resting pain. However, further investigation is needed to determine whether TCPRF’s selective efficacy is primarily associated with inflammation-related pain in SAIS. Additionally, activity-related pain is known to negatively affect rehabilitation participation and clinical outcomes in chronic musculoskeletal pain conditions. Therefore, the inability of TCPRF to effectively reduce activity-related pain should be interpreted with caution, given the limited evidence regarding its clinical effectiveness and applicability. Future studies should investigate the specific mechanisms underlying TCPRF’s selective efficacy, particularly in relation to inflammatory pain processes. Additionally, research comparing the effects of single versus multiple-session TCPRF treatments could help determine the optimal treatment protocol for improving outcomes in SAIS, especially for activity-related pain.

## 5. Conclusions

TCPRF is a needle-free, portable, noninvasive, and painless therapy that does not require sedation. Our study found TCPRF superior to sham treatment in reducing rest pain and improving SPADI scores, particularly in the total and disability subscales, at the 12th week. However, no significant differences between the TCPRF and sham groups were observed at other time points or for other outcome measures. The observed differences at only one time point and in specific measures do not allow us to conclusively establish TCPRF’s superiority over sham treatment. Given the lack of consensus on optimal TCPRF treatment parameters—such as frequency, duration, current width, voltage, frequency, and electrode placement—further research is needed to determine the most effective TCPRF configurations before arriving at a conclusive judgment. Our study may pave the way for future research aimed at establishing the optimal TCPRF therapy parameters for SAIS.

## Figures and Tables

**Table 1:** Demographic and clinical characteristics.

	Control (n = 23)	TCPRF (n = 27)	*p* Value
Age (years)	50 (43–56)	53 (47–61)	0.419 ^†^
BMI (kg/m^2^)	27.1 (23.7–30.8)	25.8 (24.8–31.2)	0.853 ^†^
Gender			
Male	5 (21.7%)	7 (25.9%)	0.730 ^‡^
Female	18 (78.3%)	20 (74.1%)	
Education level			
Primary school	7 (30.4%)	11 (40.7%)	0.754 ^‡^
Middle school	3 (13.0%)	3 (11.1%)	
High school	6 (26.1%)	8 (29.6%)	
University	7 (30.4%)	5 (18.5%)	
Dominant extremity			
Right	23 (100%)	26 (96.3%)	0.540 ^‡^
Left	0	1 (3.7%)	
Affected side			
Right	16 (69.6%)	15 (55.6%)	0.309 ^‡^
Left	7 (30.4%)	12 (44.4%)	
Duration of shoulder pain (months)	6 (5–12)	10 (5–10)	0.361 ^†^
SSPT	6.9 (5.6–7.9)	7.1 (4.8–9.0)	0.633 ^†^
AHD	10.2 (8.8–10.6)	11 (10–11)	0.063 ^†^

^†^ Mann Whitney U test; ^‡^ Fischer Chi square; Abbreviations: TCPRF: Transcutaneous Pulsed Radiofrequency; BMI: Body Mass Index; SSPT, Supraspinatus Tendon; AHD: Acromiohumeral Distance. Median (IQR) and frequency (%) are given.

**Table 2:** Shoulder pain at activity and rest and SPADI results.

	Baseline			1st Week			4th Week			12th Week		
	Control	TCPRF	*p* Value	Control	TCPRF	*p* Value	Control	TCPRF	*p* Value	Control	TCPRF	*p* Value
Activity pain (VAS)	8 (7–8)	8 (7–10)	0.223	5 (3–8)	5.5 (3–7)	0.613	7 (3–8)	4 (2–7)	0.097	6 (3–7)	3.5 (2–6.3)	0.192
Resting pain (VAS)	3 (1–6)	3 (2–4)	0.791	1 (1–3)	2 (1–3.3)	0.721	1 (0–3)	1 (1–2.5)	0.312	1 (1–3)	0 (0–2)	0.041
SPADI–Total	87 (54–105)	84 (69–107)	0.704	58 (30–94)	58.5 (41–79.3)	0.984	52 (31–96)	44.5 (20–72.5)	0.288	50 (29–82)	21 (9–66.3)	0.017
SPADI–Pain	36 (26–42)	37 (32–44)	0.192	29 (12–39)	25.5 (18–37)	0.904	29 (15–40)	24.5 (11.3–33.3)	0.489	27 (12–33)	10.5 (6.5–28.3)	0.052
SPADI–Disability	49 (30–63)	47 (35–62)	0.778	30 (18–60)	33 (20–44.5)	0.976	28 (16–57)	20.5 (10.8–41)	0.193	30 (15–30)	11 (5.8–38.8)	0.008

Abbreviations: TCPRF: Transcutaneous Pulsed Radiofrequency; VAS: Visual Analog Scale; SPADI: Shoulder Pain and Disability Index. Values are presented as median (IQR).

**Table 3:** Analgesic use in control and TCPRF groups.

	Control	TCPRF		Control	TCPRF		Control	TCPRF	
Analgesics	Bazal-1st Week	Bazal-1st Week	*p* Value	1st Week–4th Week	1st Week–4th Week	*p* Value	4th Week–12th Week	4th Week–12th Week	*p* Value
No	16 (69.6%)	19 (70.4)	0.373	19 (82.7%)	22 (81.5%)	0.889	20 (86.9%)	24 (88.9%)	0.978
Paracetamol	6 (26.1%)	6 (22.2%)		3 (13%)	3 (11.1%)		2 (8.7%)	2 (7.4%)	
NSAID	1 (4.3%)	2 (7.4%)		1 (4.3%)	2 (7.4%)		1 (4.3%)	1 (3.7%)	

Abbreviations: TCPRF: Transcutaneous Pulsed Radiofrequency; NSAID: Non-Steroidal Anti İnflammatory Drugs.

**Table 4:** Time Point Comparisons of Statistically Significant Changes in VAS, SPADI, Shoulder Range of Motion, and SSPT for TCPRF and Control Groups.

	Group	Comparison	Z Value	*p* Value
Activity Pain	Control	Baseline—1st Week	−3.370	0.001
		Baseline—4th Week	−3.433	0.001
		Baseline—12th Week	−4.044	<0.001
		4th Week—12th Week	−2.996	0.003
	TCPRF	Baseline—1st Week	−3.975	<0.001
		Baseline—4th Week	−4.008	<0.001
		Baseline—12th Week	−4.207	<0.001
Resting Pain	Control	Baseline—12th Week	−3.032	0.002
	TCPRF	Baseline—12th Week	−3.240	0.001
SPADI–Total	Control	Baseline—1st Week	−2.921	0.003
		Baseline—4th Week	−3.682	<0.001
		Baseline—12th Week	−4.075	<0.001
		4th Week—12th Week	−2.852	0.004
	TCPRF	Baseline—1st Week	−3.688	<0.001
		Baseline—4th Week	−4.178	<0.001
		Baseline—12th Week	−4.432	<0.001
		1st Week—12th Week	−3.645	<0.001
		4th Week—12th Week	−4.042	<0.001
SPADI–Pain	Control	Baseline—1st Week	−2.680	0.007
		Baseline—4th Week	−2.940	0.003
		Baseline—12th Week	−4.109	<0.001
	TCPRF	Baseline—1st Week	−3.517	<0.001
		Baseline—4th Week	−4.058	<0.001
		Baseline—12th Week	−4.319	<0.001
		1st Week—12th Week	−3.774	<0.001
		4th Week—12th Week	−3.878	<0.001
SPADI–Disability	Control	Baseline—1st Week	−3.166	0.002
		Baseline—4th Week	−3.530	<0.001
		Baseline—12th Week	−3.790	<0.001
	TCPRF	Baseline—1st Week	−3.702	<0.001
		Baseline—4th Week	−4.128	<0.001
		Baseline—12th Week	−4.292	<0.001
		1st Week—12th Week	−3.487	<0.001
		4th Week—12th Week	−3.619	<0.001
Active Flexion	TCPRF	Baseline—12th Week	−2.733	0.006
Passive Flexion	TCPRF	Baseline—1st Week	−2.820	0.005
		Baseline—12th Week	−2.733	0.006
Active Abduction	TCPRF	Baseline—4th Week	−2.691	0.007
		Baseline—12th Week	−2.721	0.007
Passive Abduction	TCPRF	Baseline—4th Week	−2.691	0.007
		Baseline—12th Week	−2.721	0.007
SSPT	Control	Baseline—1st Week	−3.214	0.001
		Baseline—4th Week	−2.970	0.003
		Baseline—12th Week	−3.200	0.001
	TCPRF	Baseline—1st Week	−2.814	0.005
		Baseline—4th Week	−3.109	0.002
		Baseline—12th Week	−3.015	0.003

Abbreviations: TCPRF: Transcutaneous Pulsed Radiofrequency; SPADI: Shoulder Pain and Disability Index; SSPT: Supraspinatus Tendon.

## Data Availability

The data associated with this article can be obtained from the corresponding authors upon reasonable request.
